# The Mural Form of Eosinophilic Esophagitis Is Accompanied by Superficial Esophageal Squamous Cell Carcinoma

**DOI:** 10.1155/2012/315428

**Published:** 2012-02-28

**Authors:** Zhao Jingsheng, Luo Yuncheng, Miao Yingye, Li Hao, Li Congyang

**Affiliations:** ^1^Department of Pathology, People's Liberation Army 152 Hospital, Pingdingshan 467000, China; ^2^Department of Thoracic Surgery, People's Liberation Army 152 Hospital, Pingdingshan 467000, China; ^3^Department of Clinical Laboratories, People's Liberation Army 152 Hospital, Pingdingshan 467000, China

## Abstract

Eosinophilic esophagitis (EE) is an increasingly recognized primary clinicopathologic disorder of the esophagus which lacks a specific etiology. Most reports on EE have been limited to the esophagus mucosa. We present a 56-year-old man with the mural form of EE and superficial squamous cell carcinoma in the esophagus. The eosinophils diffusely invaded the full-thickness of the esophagus, mainly infiltrating the muscularis, including the skeletal and smooth muscles. The lesions in the mucosa, submucosa, and adventitia were slight. Although the superficial squamous cell carcinoma was excited by an endoscopic biopsy, there were some changes in the architecture and size of the squamous epithelial cells. The changed cells also expressed the p53 protein. It appears that the eosinophils stimulated cell proliferation, followed by genetic mutations and cancer development. The patient survived with resection of the esophagus and inhaled corticosteroids.

## 1. Introduction

Eosinophilic esophagitis (EE) was first described by Furuta et al. and deemed a variant of eosinophilic gastroenteritis [1]. Since then, reports on this condition have increased. EE is an increasingly recognized primary clinicopathologic disorder of the esophagus which lacks a specific etiology [2, 3]. Its symptoms include dysphagia, vomiting, regurgitation, nausea, epigastric pain, and heartburn. Endoscopic features include rings, furrows, white specks, and a narrow caliber esophagus [4]. Most reports on EE have been limited to the esophagus mucosa. Here, we report a very rare case of a mural form of EE that is associated with esophageal superfical squamous cell carcinoma.

## 2. Case Report

### 2.1. Clinical History

**Figure 1:**
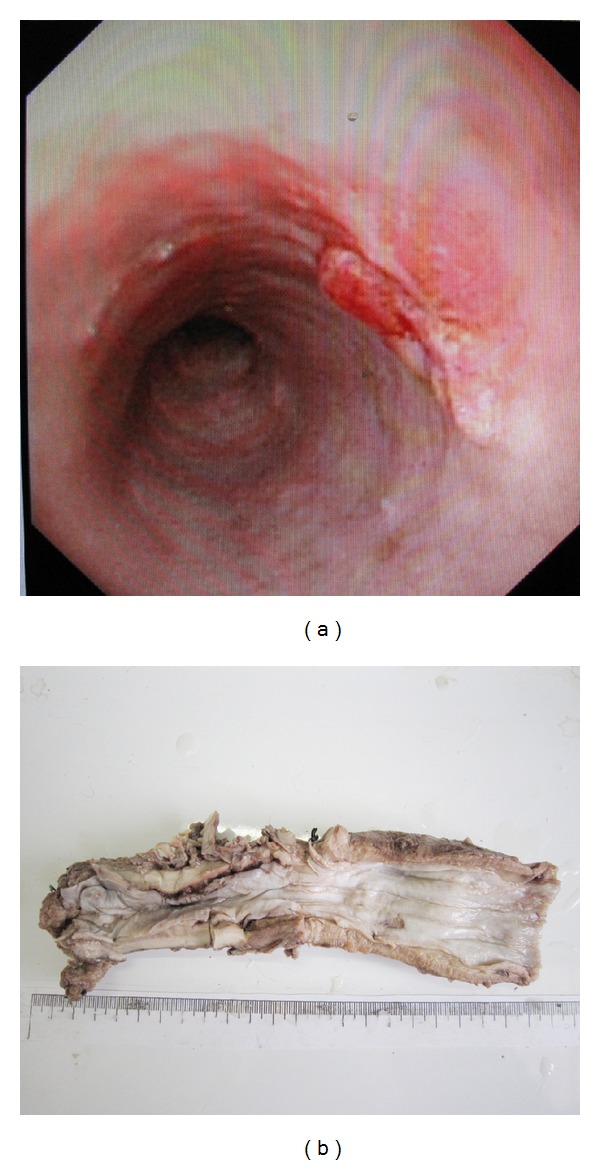
Endoscopic examination and gross presentation. (a) Presence of a white mass in the mucosa. (b) The resected esophagus showed mucosal abnormalities located 1 cm from the proximal margin. The entire esophagus was very hardened and thick.

A 56-year-old man presented as having abdominal distention with no reason for six years. Chinese medicine helped to relieve his symptoms. He had no other complaints, although six months prior, his skin had started itching, and three weeks prior he felt unable to successfully swallow hard food but could swallow semifluid food. He also had no history of asthma or allergies. The man went to the local hospital and had a gastroscopic examination, which showed a mass of about 1.3 cm × 1.2 cm in size (Figure 1(a)), located on the posterior wall 34 cm from the incisors. Mucosa, in the cardiac stomach, the body/fundic stomach, the pyloric stomach, and the duodenal bulb were relatively normal. An esophageal biopsy was performed and squamous cell carcinoma was diagnosed, requiring the patient to undergo surgery. During the operation, the surgeon found that the thoracic portion of the esophagus was thickened and hard, but the stomach was normal. So the surgeon excised the middle and lower esophagus, cardia, and part of epiploon.

### 2.2. Macroscopic Examination

The esophagus was resected, which was 20 cm in length and 3 cm in circumference. The entire esophagus was very hardened and thick. There was mucosal erosion of about 1.2 cm × 1.1 cm in size (Figure 1(b)), located 1 cm from the proximal margin. Two lymph nodes were detected in the lateral esophagus.

### 2.3. Microscopic Examination

#### 2.3.1. Esophageal Biopsy Specimen

**Figure 2:**
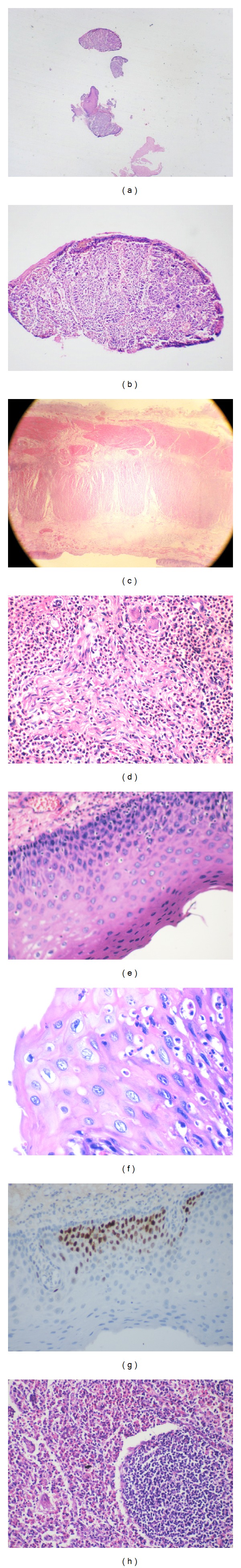
Histologic examination. (a) Presence of three lesions between the normal tissue. (b) Superficial squamous cell carcinoma with infiltration of eosinophils into the tumor cells and lamina propria. (c) Presence of mucosal coloboma. (d) The eosinophils infiltrated the muscle fiber and nerve plexus. (e) Presence of basal hyperplasia. (f) Changes in polarity and size of the cell and nuclei. (g) Basal parabasal cells expressed p53. (h) Eosinophils invaded the lymph nodes.

Five tissues were biopsied (Figure 2(a)). Two tissues showed normal stratified squamous epithelia. However the squamous epithelia in the other three tissues exhibited cellular and architectural abnormalities and displayed chaos in polarity and arrangement. The cells also had enlarged and hyperchromatic nuclei. In some areas, the cells penetrated through the epithelial basement and invaded the lamina propria (Figure 2(b)). Under a high-power field, we also saw that lots of eosinophils had infiltrated the tumor cells and lamina propria.

### 2.4. Esophageal Resection Specimen

After esophageal resection, there were mucosal coloboma and no remnants of squamous cell carcinoma (Figure 2(c)). The striking feature is that eosinophils diffusedly invaded the mucosa, submucosa, muscularis, and adventitia (Figure 2(c)). The lesions in the mucosa, submucosa, and adventitia were slight. The eosinophils mainly infiltrated the muscularis, including the skeletal muscle and smooth muscle, as well as the muscle fiber. The notable characteristic was the eosinophilic abscess in the muscularis (Figure 2(d)). The nerve plexus in the muscularis was also separated by eosinophils (Figure 2(d)). However, the nerve plexus in the submucosa and adventitia were less influenced by the eosinophils, and there were no apparent eosinophils in the blood vessel of all layers. Although there was no remnant squamous cell carcinoma, basal cell hyperplasia is common (Figure 2(e)) and thus altered architecture and abnormalities in cytology could be seen (Figure 2(f)). In some areas, about 2% the basal cell and parabasal cells expressed p53 (Figure 2(g)). Although there were eosinophils in the esophageal gland stroma, the acinus was not destroyed by the eosinophils. Many eosinophils filled the sinus of the esophagus lymph node (Figure 2(h)). Although there were lympholeukocytes in the epiploon, no eosinophils were present.

### 2.5. Laboratory Tests

**Figure 3:**
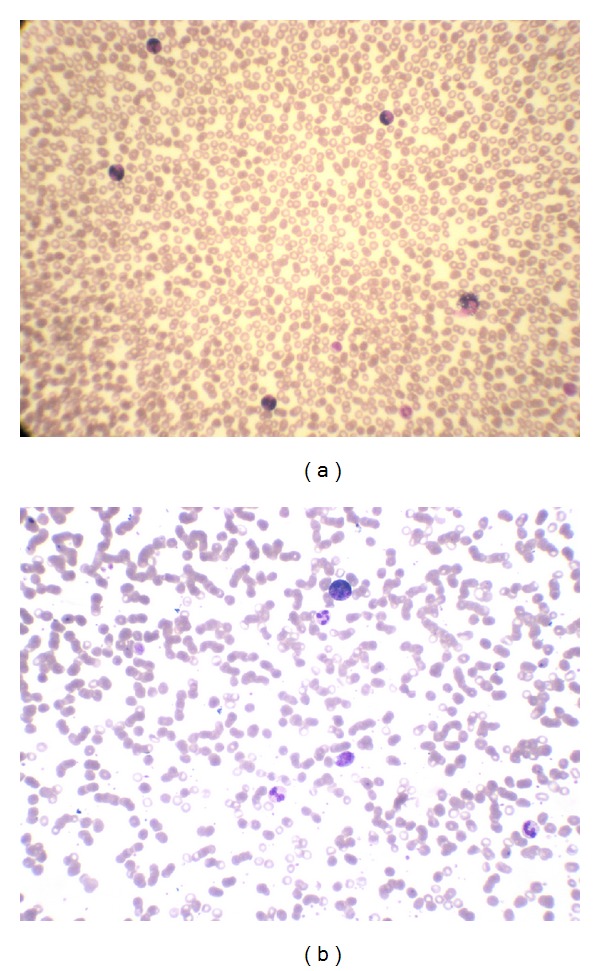
Blood smear (Wright Giemsa). (a) The examination before surgery showed 52% eosinophils. (b) The examination after surgery showed 10% eosinophils.

During hospitalization, laboratory tests were done and showed an eosinophil count of 52% (absolute number was 3.95 × 10^9^). A subsequent blood smear examination showed 50% (absolute number was 3.70 × 10^9^) eosinophils (Figure 3(a)). Although the esophagus was resected, the eosinophil count decreased to 10% (absolute number was 0.69 × 10^9^) (Figure 3(b)). After the patient was treated with inhaled corticosteroids, the eosinophil count returned to normal.

## 3. Discussion

Eosinophilic esophagitis (EE) is defined as a primary clinicopathological disorder of the esophagus. It is characterized by esophageal and/or upper gastrointestinal tract symptoms associated with esophageal mucosal biopsy specimens containing ≥15 intraepithelial eosinophils/HPF (high-power field) in one or more biopsy specimens. It is also characterized by the absence of pathologic gastrointestinal reflux disease, as evidenced by normal pH monitoring of the distal esophagus, or lack of response to high-dose proton pump inhibitor medications [4]. In our case, the eosinophils diffusedly infiltrated the full-thickness of esophageal wall, and a diagnosis of eosinophilic esophagitis was made. Therapy with inhaled corticosteroids reduced the eosinophil count to normal.

EE should be distinguished from gastroesophageal reflux disease (GERD). In our case, the patient had no symptoms of heartburn and regurgitation. Eosinophils diffusely invaded the middle and lower esophagus, generally exceeding 15/HPF. The blood eosinophils returned to normal when the patient was treated with inhaled corticosteroids, so GERD was excluded. Eosinophilic gastroenteritis (EGE) was also a possibility as this condition involves the esophagus. However, although the patient had peripheral blood eosinophilia, he had no complaints of abdominal pain, nausea, weight loss, or diarrhea. In addition, there were no eosinophils in the epiploon, and the surgeon found that the stomach was soft, so the diagnosis of EGE was removed as well. Eosinophilic fasciitis also involves the esophagus. However, the antinuclear antibody, anti-DNA antibody, and rheumatoid factor were normal, so a diagnosis of eosinophilic fasciitis was not made.

The cause of EE is still uncertain. Fifty to eighty percent of EE patients suffer from other allergic conditions [5], and antiallergic therapies have resulted in significant clinical and histological remission of the disease [4, 6, 7]. Therefore, it has been suggested that EE is an inflammatory response to a specific diet or to aeroallergens. However, there is a small proportion of EE patients who have no identifiable allergic sensitizations [8, 9]. In the present case, the patient had no history of asthma or allergies, such as those to food, drug, or pollen. Although the causes underlying EE may be different, allergic and nonallergic esophagitis may have similar effector pathways [10, 11]. Blanchard et al. identified a striking EE transcript signature involving approximately 1% of the human genome. This transcriptome is remarkably conserved in patients despite differences in age, sex, and allergic status. The authors also found that the downstream effector phase of the disease is conserved between atopic and nonatopic variants of EE [11]. Humbert et al. showed that atopic and nonatopic patients have the same expression of cytokine mRNA expression in lung tissue of asthma [11, 12]. These studies indicate that the therapy for atopic and nonatopic patients maybe had the same measure [11]. Our case strongly supports this point. Although our patient showed no identifiable allergic sensitizations, his condition was sensitive to inhaled corticosteroids.

Eosinophils can be present in malignant tumors. As we know, malignant tumor cells can release the cytokines, eotaxin, IL-5, and eosinophil chemotactic factor of anaphylaxis (ECF-A), which stimulates the recruitment of eosinophils from circulation into the tumor site. However, there is no case report about EE-associated superficial squamous cell carcinoma of the esophagus in English literature. In our case, the patient's clinical history showed that EE existed first, and squamous cell carcinoma confined to the mucosa developed later. The relationship between EE and esophageal squamous cell carcinoma is unclear. In EE, basal cell hyperplasia correlates with the density of intraepithelial eosinophils [2]. In the present case of basal cell hyperplasia, cells showed chaos in arrangement as well as variation in shape and size of nuclei. The nuclear-cytoplasmic ratio increased as well, and the cells in the basal and parabasal layers expressed the p53 protein. It is possible that inflammation provoked cell proliferation, followed by genetic mutations, and the development of cancer.

In our case, the patient could not successfully swallow hard food. This symptom may be related to injury of the muscle and nerve plexus of the muscularis. After the surgery was complete and inhaled corticosteroids were taken, there were no further complaints of abdominal distention and itching skin. The patient survived with no recurrence of his condition.
